# Nationwide Analysis on Intentional Indoor and Outdoor Tanning: Prevalence and Correlates

**DOI:** 10.3390/ijerph191912309

**Published:** 2022-09-28

**Authors:** Katharina Diehl, Eckhard W. Breitbart, Rüdiger Greinert, Joel Hillhouse, Jerod L. Stapleton, Tatiana Görig

**Affiliations:** 1 Department of Medical Informatics, Biometry and Epidemiology, Friedrich-Alexander-Universität Erlangen-Nürnberg (FAU), 91054 Erlangen, Germany; 2 Arbeitsgemeinschaft Dermatologische Prävention, 21614 Buxtehude, Germany; 3 College of Public Health, East Tennessee State University, Johnson City, TN 37614, USA; 4 Department of Health, Behavior & Society, College of Public Health, University of Kentucky, Lexington, KY 40506, USA

**Keywords:** tanning beds, sunbeds, sunbathing, intentional tanning, outdoor tanning, determinants

## Abstract

Outdoor and indoor tanning are considered as risk factors for the development of skin cancer. The aims of this nationwide representative study were to quantify both behaviors in a sample with a wide age range, to identify those showing both behaviors and to explore and compare determinants of both behaviors. We used data from the fifth wave (2019) of the National Cancer Aid Monitoring (NCAM). We surveyed the representative sample including 4000 individuals, aged 16–65 years, living in Germany. Data were collected through telephone interviews. In addition to descriptive statistics, we used logistic regression analyses to identify determinants. The one-year-prevalence of tanning bed use was 7.5%, while 31.9% tanned (very) often intentionally outdoors in at least one situation (weekdays, holidays, and weekends). A total of 3.2% reported both risk behaviors. Regression analyses revealed that tanning bed use is associated with employment, an increased number of naevi, and lack of risk awareness. Intentional outdoor tanning was associated with male sex, younger age, past tobacco use, and low risk awareness of UV radiation. Our findings suggest that only a minority of subjects showed both risk behaviors. This implies that individuals seem to perform either one behavior or the other. In addition, the associated determinants differed between both behaviors, implying that specific preventive measures tailored to address to each tanning behavior are needed.

## 1. Introduction

While the incidence of skin cancer is increasing among the white population worldwide [1,2,3] and one in every three diagnosed cancers is a skin cancer [4], tanned skin is perceived as desirable in many Western countries [5,6,7]. There are two ways to achieve tanned skin besides sunless tanning products, such as spray tanning, lotions, and creams [8]: (natural) solar ultraviolet radiation (UVR) and artificial UVR through the use of indoor tanning beds or sunlamps. Both types of these tanning practices were classified as “carcinogenic to humans” by the International Agency for Research on Cancer (IARC) in 2009 [9].

UVR is the main environmental risk factor for the development of malignant melanoma, basal cell carcinomas, and squamous cell carcinomas [9,10]. Despite this, the desire for tanned skin leads people to intentionally expose themselves to the sun. Representative and population-based studies in the general population have shown that approximately one out of ten people often intentionally tan outdoors (e.g., 9.5% usually/always tan outdoors [11], 10.1% showed >30 days of sun exposure per year [12], and 14.1% used every opportunity to tan outdoors [13]). Regarding outdoor tanning and sex, previous studies led to mixed findings; however, the tendency is that women are more likely to tan outdoors compared to men [11,14]. In addition, tanning outdoors is associated with younger age [11].

Exposure to artificial tanning devices (i.e., tanning beds, sunbeds, solaria) has been shown to be associated with an increased risk of developing skin cancer, particularly melanoma [9,15,16]. A recent literature review reports the global past-year prevalence of tanning bed use worldwide is 10.4% among adults and 6.7% among adolescents [17]. Besides age, indoor tanning was shown to be associated with female sex in previous studies [17,18]. In addition to enhancing attractiveness, tanning beds are used for relaxation, pre-tanning for holidays, light exposure, warmth, and vitamin D supplementation [19,20].

National studies have typically reported the prevalence of either outdoor tanning or tanning bed use, and only a limited number of studies included both aspects of UV-exposure [12,13,21,22,23,24]. This represents an important gap in our understanding because combining both is a high-risk UV-exposure behavior, which likely would increase skin cancer risk. Additionally, understanding whether intentional indoor and outdoor tanning are practiced separately or combined will be important for developing more efficacious prevention interventions. Therefore, we conducted a nationwide representative study assessing numerous potential determinants of intentional outdoor tanning and tanning bed use in a sample with a wide age range (16 to 65 years old).

This study aims to deepen the understanding on this topic by (1) quantifying intentional outdoor tanning and tanning bed use in 16- to 65-year-olds living in Germany; (2) exploring and comparing determinants individually for both risk behaviors, and (3) identifying and describing those showing both risk behaviors, i.e., current tanning bed use and frequent outdoor tanning.

## 2. Methods

### 2.1. Study Setting

We used cross-sectional data from the fifth wave of the German representative National Cancer Aid Monitoring (NCAM), which is conducted by the authors. Between October and December 2019, 4000 individuals aged 16 to 65 years participated in computer-assisted telephone interviews (CATI). Random sampling involved a two-stage procedure, as described elsewhere [20,25]. Response rate was calculated based on criteria of the American Association on Public Opinion Research (AAPOR; Response Rate [RR] 3 = 28.9%). All subjects provided informed consent to participate in this study. Data are weighted on age, sex, and education based on the German Microcensus to be representative for the German population.

### 2.2. Instruments and Measures

#### 2.2.1. Outcome Variables

Two outcome variables were considered in the present study. First, we assessed current tanning bed use, defined as at least one tanning session within the last 12 months. In addition, the number of tanning sessions within the last 12 months was assessed [20]. Second, we gathered information on intentional outdoor tanning. Since the frequency of intentional tanning might differ depending on the context, we used three questions: “How often do you go in the sun in order to get a tan during your holidays?”; “In summer, how often do you go in the sun in order to get a tan on the weekend?”; “In summer, how often do you go in the sun in order to get a tan on a typical workday?”. Response categories were very often, often, sometimes, rarely, and never. We adapted questions suggested in previous research [26,27]. Cognitive interviewing (*n* = 15) was used to pretest these questions. For analyses, a dummy variable was used to contrast frequent outdoor tanning (i.e., individuals who tanned very often or often on all above-mentioned situations) and non-frequent outdoor tanning (i.e., those who tanned sometimes, rarely or never on these situations). 

#### 2.2.2. Covariates

Basic **socio-demographic characteristics** were assessed including sex, age, immigrant background (yes vs. no), school education (low, i.e., those who are still at school, have no school-leaving qualification, or have secondary modern school qualification, medium, i.e., secondary school certificate, high, i.e., higher education entrance qualification), relationship status (having a partner: yes vs. no), and employment (none, part-time, full-time).

The interviews also included questions related to **lifestyle characteristics**: (1) smoking (current, past, never); (2) use of e-cigarettes (current, past, never); (3) paying attention to a healthy diet (very much/much vs. partly/not much/not at all); and (4) paying attention to sufficient physical activity (very much/much vs. partly/not much/not at all). 

Additional questions related **risk factors for skin cancer**: (1) self-reported skin type according to the classification suggested by Fitzpatrick, which were grouped into skin types I-II, III, and IV-VI; (2) individual history of sunburn < 15 years of age (often vs. not often/do not know); (3) number of naevi (≥40 vs. <40); (4) family history of malignant melanoma (yes vs. no/do not know); and (5) individual’s history of malignant melanoma (yes vs. no/do not know).

We also assessed the agreement of participants with the following statements related to **risk awareness of tanning beds and UVR:** “Each sunburn leaves permanent damage to the skin”; “Regular use of tanning beds causes premature skin aging”; “Regular tanning bed use increases skin cancer risk”. Three response categories were predefined as: rather agree, rather disagree, and don’t know.

### 2.3. Statistical Analysis

Data were weighted by age, sex, and education based on the German Microcensus to reach a nationally representative sample. Descriptive analyses and chi-squared tests were used to explore and compare the distribution of covariates. Additionally, logistic regression analyses, including significant covariates in bivariate analyses, were performed. For both outcome variables, five regression models were applied based on thematic considerations: (I) including only sociodemographic variables, (II) including only lifestyle characteristics, (III) including only skin-related risk factors, (IV) including only UV-related risk awareness variables, and (V) the final model, including significant covariates in models I–IV. All analyses were performed using SPSS version 25 (IBM Corporation, Armonk, NY, USA) with a predefined level of significance of *p* < 0.05.

## 3. Results

The study sample (*n* = 4000) was divided nearly equally by sex (Table 1). Nearly half of the participants (44.8%) belonged to the group of 46–65 year-olds, had no immigrant background (85.8%), were in a relationship (67.8%), and were part- or full-time employed (76.0%). 

With regard to lifestyle characteristics, the majority of respondents were past or never users of tobacco cigarettes (74.8%) or e-cigarettes (92.9%), paid (very) much attention to a healthy diet (60.6%) and sufficient physical activity (59.3%). Regarding the prevalence of risk factors in the study sample, 40.7% reported having fair skin (type I–II), 7.4% reported often being sunburned before age 15, 32.6% had >40 naevi, 11.6% reported malignant melanoma in first-grade relatives, and 4.0% were diagnosed with malignant melanoma themselves. Approximately three quarters of participants were aware of the potential risks of UVR based on the three included items (Table 1).

### 3.1. Intentional Outdoor Tanning

Considering all three opportunities for intentional outdoor tanning, 13.8% reported to tan (very) often in all of the scenarios (on holidays, weekends, and workdays). While 7.4% tanned outdoors on two out of three situations, 10.8% tanned only on one. The majority of our study sample (68.1%) tanned outdoors rarely or not at all. 

Positive associations between intentional outdoor tanning and male sex (OR = 0.68 for females, OR = 1.00 for males; *p* < 0.001), past smoking (OR = 1.37; *p* = 0.012), and low UV-related risk awareness remained significant in multivariate logistic regression analyses (Table 2, Model V). Participants aged 46–55 years (OR = 0.33; *p* < 0.001) and 56–65 years (OR = 0.17; *p* < 0.001) were less likely to tan outdoors in summer compared to younger participants in our study. A higher likelihood of intentional outdoor tanning was identified in respondents aged 26–35 years (OR = 1.39; *p* = 0.016). 

### 3.2. Tanning Bed Use

In total, 7.5% of study participants reported tanning bed use within the last 12 months. The mean number of tanning sessions within the last 12 months among participants who reported any indoor tanning bed use was 5.78 (SD: 6.98, min: 1, max: 53). In multivariate logistic regression analyses, the use of tanning beds was positively associated with being part- or full-time employed (OR = 2.45 and OR = 2.08, respectively; both *p* < 0.01), past smoking (OR = 1.46; *p* = 0.022), or current e-cigarette use (OR = 1.73; *p* = 0.015; Table 3, Model V). Respondents with > 40 naevi (OR = 1.31; *p* = 0.033), as well as those with low risk awareness were also more likely to use tanning beds compared to their counterparts.

### 3.3. Combination of Outdoor and Indoor Tanning

**Figure 1:**
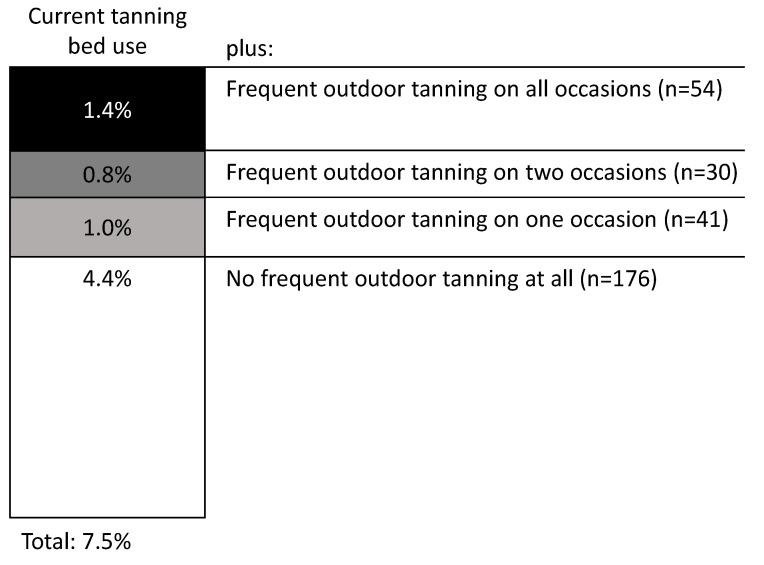
Combination of current tanning bed use with outdoor tanning.

Analyzing the combination of indoor and outdoor tanning, we found that 3.2% of the participants combined frequent outdoor tanning with current tanning bed use. In this group, 1.8% were current tanning bed users and tanned outdoors (very) often at one or two situations (Figure 1). The remaining 1.4% (*n* = 54) currently used tanning beds and intentionally tanned outdoors (very) often on all three situations. Of these participants, 24 (44%) were women (*p* = 0.497), 28 (51%) were aged between 16 and 35 years (*p* = 0.023), 12 (22%) reported an immigration background (*p* = 0.118), and 30 (55%) were in a relationship (*p* = 0.041). This high-risk group was nearly equally distributed to the groups of skin type: I–II (*n* = 21, 39%), III (*n* = 17, 32%), IV-VI (*n* = 16, 30%, *p* = 0.943). 

*n* = 4000 individuals 16–65 years of age who participated in National Cancer Aid Monitoring in 2019; data is weighted by sex, age, education level and state of residence.

Current tanning bed use = use within the last 12 months; frequent outdoor tanning = (very) often intentional outdoor tanning.

Situations for intentional outdoor tanning were weekend, weekday, and holiday. Proportions may not always sum up due to rounding

## 4. Discussion

We aimed to describe in detail current tanning bed use and frequent outdoor tanning to compare determinants of both UV exposure behaviors and to quantify the proportion of those engaged in both risk behaviors to a high degree. In our study, 3.2% of participants reported both behaviors (i.e., current tanning bed use and (very) often intentional outdoor tanning on at least one situation), while an additional 4.4% were tanning bed only users, and additional 28.7% (very) frequently tanned on at least one outdoor situation. This suggests the existence of three divergent types of “tanners” that need attention regarding specific prevention measures.

The generalizability of this distinction among these three types of risky tanners among other samples should be investigated in future studies. It would also be worthwhile to investigate factors that distinguish these groups such as potential differences in tanning motives. Previous nationwide studies focusing on both tanning behaviors differed in their approach compared to our study. Shoemaker et al. [11] used a general variable on having ever used indoor tanning facilities to investigate associations with intentional outdoor tanning. They found that people having used a tanning bed, at least once, showed a higher prevalence in intentional outdoor tanning. Haluza et al. [12,28] assessed the habit of general tanning bed use, as a covariate in the analyses of sun exposure (and vice versa) and found a significant positive association between both behaviors. Hansen et al. [13] combined questions on sunbathing and current sunbed use in an index and treated this index for the dependent variable in their analyses. By using a more concrete variable on tanning bed use (current use instead of ever or general use) and by including different situations for intentional tanning, we are able to contribute to the current state of research.

Overall, 7.5% of participants reported current use of tanning beds. This finding is in line with previous international research and reflects the global decrease in tanning bed use over the last decade [17,18,20,28,29]. While tanning bed use can be performed all year long (additional analysis of the SUN-Study 2012 showed no significant differences in prevalence between survey waves in summer and winter [19]), sunbathing is mainly limited to the summer months. Exceptions may be those traveling to sunny destinations in winter. Overall, 13.8% of study participants intentionally tanned outdoors often or very often on holidays, weekends, and workdays, which is comparable to the numbers reported in past research. Shoemaker et al. [11] found a lower prevalence (9.5% of individuals tanning usually/always intentionally outdoors), but included a slightly older age group than in our study (18–86 years vs. 16–65 years). The age differences may explain the reduced frequency since sunbathing is more frequent in younger individuals as shown in our study, in Shoemaker et al., and in others [11,12]. The prevalence in our study is also comparable to that of other studies using different items and questions to assess intentional outdoor tanning [12,13]. The unique aspect of our study is the ability to distinguish outdoor tanning using different situations (here: weekday, weekends, and holiday). While Haluza et al. [12] assessed the number of days of sunbathing, duration, and sunbathing during midday hours, we found that people often use more than one opportunity to sunbathe—an observation which should be considered when developing future prevention measures. Of those engaged in (very) frequent intentional outdoor tanning (in total: 31.9%), more than one third (13.8% of total population) tanned (very) often on all three situations (i.e., weekday, weekends, and holiday), which shows that skin cancer prevention and education needs to focus on different free time opportunities to tan outdoors.

Our study shows that tanning bed users and those who frequently tan outdoors are very different in terms of their sociodemographic characteristics. In multivariate analyses, tanning bed use was only associated with employment: part- and full-time employed participants were more likely to use tanning beds. This may be due to the fact that indoor tanning has an associated cost, and that those who are working all day long are not able to tan in the natural sun. Thus, the perceived advantage of tanning beds over outdoor tanning might be the flexibility of usage. Similar associations were also reported in earlier studies [19]. Although higher usage of tanning beds has been found in females [17,18], our study did not reveal this association. Trend analysis in Germany has shown that sex differences in indoor tanning have balanced out over the last years [20]. 

With respect to outdoor tanning, however, sex-related differences in prevalence were observed. We found a higher likelihood for intentional outdoor tanning among men compared to women, although previous studies mainly showed a higher prevalence for women [11,14] or did not find any significant differences [12]. This difference might be related to a different operationalization of outdoor tanning. While previous studies assessed either number of days participants sunbathed outdoors [12], or general intentional tanning frequency with no differentiation according to the context [11,14], we separately asked for tanning during holidays, on weekends, and on workdays in our study.

In our study, tanning bed use and intentional outdoor tanning also differed with respect to their associations with skin characteristics. In multivariate analyses, tanning bed use was positively related to naevi number, while for intentional outdoor tanning, there was no significant association. While intentional outdoor tanning was associated with skin type in bivariate analysis, no such association could be observed for tanning bed use. Previously, however, a higher likelihood of tanning bed use for people with darker skin type was reported [18,28,30]. This was also found in previous studies from Germany [19,31]. Overall, the association between tanning bed use and skin characteristics seems to be ambiguous; therefore, it is reasonable to address all skin characteristics in public health measures focusing on the prevention of sunbed use.

Both behaviors were positively associated with smoking history. Similar associations have been reported in prior studies [28,29]. In contrast, tanning bed use and intentional outdoor tanning seem to be decoupled from behaviors associated with a healthy lifestyle (i.e., healthy diet and physical activity).

Furthermore, both UV-exposure behaviors were associated with low risk awareness of potential consequences of tanning bed use and UVR. This shows that enhanced education on the potential risks of tanning bed use and UVR in general and its effects on the skin is still needed. However, previous research shows that knowledge and information alone do not guarantee a change in health-related behavior. Behaviors and behavioral changes are strongly influenced by one’s social environment and culture [32]. For instance, the model on social determinants suggested by Dahlgren and Whitehead [33] describes four different aspects that all can have an impact on health and health behaviors: besides individual characteristics such as age and sex, individual lifestyle, social networks as well as the socioeconomic, cultural, and physical environment can influence health and health-related behavior. Therefore, behavioral interventions also need to consider social context and life circumstances as well as—with regard to tanning bed use—political and economic aspects. 

Our study shows that in older participants (46–65 years), the prevalence of tanning bed use was higher than that of outdoor tanning. This is in contrast to participants < 45 years, who were more likely to tan outdoors. This finding underlines that prevention and education about the risks of outdoor tanning should specifically focus on individuals ≤ 45. It also further emphasizes that, despite previous campaigns have mainly focused on adolescents and young adults, additional public health messages aimed at reducing the use of tanning bed should also target people > 45 years. 

### Limitations

Although our study used a large representative sample with a wide age range and a comprehensive set of covariates, our results should be interpreted considering some potential limitations. First, our study is based on cross-sectional data, which does not allow drawing conclusions on causality. Second, the data are self-reported, which means that we cannot exclude potential social desirability and recall bias. However, previous studies on this topic [34], as well as our intensive cognitive pretesting showed that these aspects play only a minor role. To minimize potential nonparticipation bias, the introduction to the interview was phrased more generally, stating that the interview’s focus is on health and lifestyle, instead of pronouncing tanning behavior. In addition, we performed non-responder analysis. Third, our covariates on risk awareness focused mainly on risk awareness regarding tanning bed use. Therefore, we cannot conclude that these are a measure for risk awareness regarding solar UVR. Fourth, our study only focused on German population. It would be interesting to have more comparable data from other European countries to identify whether differences between countries exist. Regarding regional analyzes for Germany, we could not find any differences in tanning bed use and outdoor tanning (results not shown). Fifth, we did not include information on use of sun protection measures (e.g., use of sunscreen) in this survey wave.

## 5. Conclusions

Only three out of a hundred people aged 16 to 65 years living in Germany combined current tanning bed use with (very) frequent sunbathing in summer. However, 7.5% were current users of tanning beds and 31.9% sunbathed (very) frequently in the summer to different situations. Our findings suggest two different prevention strategies that should be investigated in future studies: (1) Focus on the general population by rising awareness about potential negative health consequences of tanning bed use and educating and informing about appropriate sun protection, maximal sun exposure based on individual skin type, and health risks of UVR. (2) Focus on specific target groups. The effort to reduce tanning bed use in Germany should specifically focus on the working population and give attention to all age groups, including both men and women, while the target group that should be made aware about the risks of artificial and natural UV exposure is particularly the younger generation.

## Figures and Tables

**Table 1:** Tanning bed use and (very) frequent intentional outdoor tanning in the German population (aged 16 to 65 years) by individual characteristics.

			Current Tanning Bed Use		(Very) Frequent Intentional Outdoor Tanning
	%	%	*p*-Value	%	*p*-Value
Total		7.5		13.8	
Sociodemographic characteristics					
Sex			0.693		**<0.001**
Female	49.2	7.7		**11.2**	
Male	50.8	7.4		**16.3**	
Age group			**<0.001**		**<0.001**
16–25 years	16.0	**6.4**		**17.1**	
26–35 years	19.7	**7.8**		**23.4**	
36–45 years	19.4	**7.7**		**21.3**	
46–55 years	22.5	**11.2**		**6.7**	
56–65 years	22.3	**4.3**		**3.5**	
Immigrant background			**0.009**		0.961
No	85.8	**7.1**		13.9	
Yes	14.2	**10.3**		13.8	
School education			**<0.001**		0.081
Low	20.0	**5.0**		13.7	
Medium	37.4	**5.6**		11.9	
High	42.6	**9.2**		14.8	
Partnership			**0.010**		0.120
Yes	67.8	**6.9**		13.3	
No	32.2	**9.2**		15.1	
Employment			**<0.001**		**<0.001**
None	24.0	**3.6**		**9.7**	
Part-time	19.9	**8.7**		**10.8**	
Full-time	56.1	**9.3**		**16.4**	
Lifestyle characteristics					
Smoking			**<0.001**		**0.001**
Current	25.2	**9.2**		**15.9**	
Past	24.9	**9.9**		**15.9**	
Never	49.9	**5.5**		**11.6**	
E-cigarette use			**<0.001**		**0.001**
Current	7.1	**12.3**		**15.2**	
Past	15.3	**11.8**		**18.5**	
Never	77.6	**6.3**		**12.8**	
Paying attention to a healthy diet			0.716		0.561
(Very) much	60.6	7.4		13.5	
Partly/not much/not at all	39.4	7.8		14.2	
Paying attention to sufficient physical activity			0.481		0.152
(Very) much	59.3	7.8		14.4	
Partly/not much/not at all	40.7	7.2		12.8	
Skin-related risk factors					
Skin type			0.721		**0.011**
I–II	40.7	7.2		**13.9**	
III	31.6	7.6		**11.8**	
IV–VI	27.7	8.0		**16.1**	
Often sunburn before age of 15			0.391		0.951
Yes	7.4	8.8		13.9	
No/don’t know	92.6	7.4		13.8	
More than 40 naevi			**<0.001**		**0.031**
Yes	32.6	**9.8**		**15.5**	
No	67.4	**6.5**		**13.0**	
Family history of malignant melanoma			**<0.001**		**0.005**
Yes	11.6	**12.6**		**18.0**	
No/don’t know	88.4	**6.9**		**13.2**	
History of malignant melanoma			**<0.001**		0.873
Yes	4.0	**14.8**		14.2	
No	96.0	**7.2**		13.8	
UV-related risk awareness					
“Each sunburn leaves a permanent damage in the skin”			**<0.001**		**<0.001**
Rather agree	77.1	**6.5**		**12.4**	
Rather disagree	19.3	**11.9**		**18.3**	
Don’t know	3.6	**7.7**		**19.6**	
“Regular use of tanning beds causes premature skin aging”			**<0.001**		**<0.001**
Rather agree	78.8	**5.7**		**12.5**	
Rather disagree	17.4	**15.6**		**19.7**	
Don’t know	3.8	**9.2**		**13.8**	
“Regular tanning bed use increases the skin cancer risk”			**<0.001**		**<0.001**
Rather agree	74.6	**4.8**		**11.6**	
Rather disagree	20.5	**18.0**		**22.2**	
Don’t know	4.9	**6.2**		**12.3**	

*n* = 4000 individuals 16–65 years of age who participated in National Cancer Aid Monitoring in 2019; data is weighted by. sex, age, education level and state of residence. *p*-values are based on Chi^2^-statistics. Bold font: results with significance of *p* < 0.05.

**Table 2:** Determinants of (very) frequent intentional outdoor tanning based on logistic regression analyses.

	Model I	Model II	Model III	Model IV	Model V
	OR [95% CI]	OR [95% CI]	OR [95% CI]	OR [95% CI]	OR [95% CI]
Sociodemographic characteristics					
Sex					
Female	**0.68 [0.55–0.83]**				**0.68 [0.56–0.82]**
Male	Ref.				Ref.
Age group					
16–25 years	Ref.				Ref.
26–35 years	**1.41 [1.04–1.89]**				**1.39 [1.06–1.82]**
36–45 years	1.27 [0.94–1.71]				1.27 [0.97–1.68]
46–55 years	**0.32 [0.22–0.46]**				**0.33 [0.23–0.46]**
56–65 years	**0.17 [0.11–0.27]**				**0.17 [0.11–0.26]**
Employment					
None	Ref.				
Part-time	0.92 [0.66–1.28]				
Full-time	1.26 [0.97–1.64]				
Lifestyle characteristics					
Smoking					
Current		**1.34 [1.05–1.71]**			1.21 [0.94–1.57]
Past		**1.29 [1.02–1.64]**			**1.37 [1.07–1.76]**
Never		Ref.			Ref.
E-cigarette use					
Current		1.02 [0.70–1.48]			0.89 [0.60–1.31]
Past		**1.35 [1.05–1.74]**			1.12 [0.85–1.47]
Never		Ref.			Ref.
Skin-related risk factors					
Skin type					
I–II			Ref.		
III			0.83 [0.66–1.04]		
IV–VI			1.23 [0.99–1.53]		
More than 40 naevi					
Yes			1.20 [0.90–1.46]		
No			Ref.		
Family history of malignant melanoma					
Yes			**1.38 [1.06–1.80]**		1.14 [0.86–1.51]
No/don’t know			Ref.		Ref.
UV-related risk awareness					
“Each sunburn leaves a permanent damage in the skin”					
Rather agree				Ref.	Ref.
Rather disagree				1.13 [0.88–1.44]	1.05 [0.82–1.34]
Don’t know				**1.89 [1.16–3.10]**	**1.67 [1.02–2.74]**
“Regular use of tanning beds causes premature skin aging”					
Rather agree				Ref.	
Rather disagree				1.07 [0.81–1.40]	
Don’t know				0.87 [0.49–1.55]	
“Regular tanning bed use increases the skin cancer risk”					
Rather agree				Ref.	Ref.
Rather disagree				**1.99 [1.55–2.56]**	**1.84 [1.46–2.32]**
Don’t know				0.90 [0.53–1.53]	0.84 [0.51–1.38]
Nagelkerke’s r^2^	0.127	0.010	0.009	0.028	0.140
*n*	3801	3979	3947	3974	3959

*n* = 4000 individuals 16–65 years of age who participated in National Cancer Aid Monitoring in 2019; data is weighted by sex, age, education level and state of residence. Dependent variable: I (Very) frequent intentional outdoor tanning holiday, on workdays and on weekends during last summer often or very often (1 = yes, 0 = no); Regression models only included variables that were significant in bivariate analyses. Model I included sociodemographic characteristics, Model II included lifestyle characteristics, Model III included skin-related risk factors, Model IV included variables on UV-related risk awareness as independent variables. Model V included only variables that were significant in preceding regression model. Bold font: results with significance of *p* < 0.05; OR: Odds Ratio; 95% CI: 95% confidence interval, Ref.: Reference Category.

**Table 3:** Determinants of current tanning bed use based on logistic regression analyses.

	Model I	Model II	Model III	Model IV	Model V
	OR [95% CI]	OR [95% CI]	OR [95% CI]	OR [95% CI]	OR [95% CI]
Sociodemographic characteristics					
Age group					
16–25 years	Ref.				
26–35 years	0.91 [0.56–1.48]				
36–45 years	1.06 [0.66–1.71]				
46–55 years	1.44 [0.92–2.27]				
56–65 years	0.65 [0.39–1.11]				
Immigrant background					
No	Ref.				Ref.
Yes	**1.53 [1.07–2.17]**				1.06 [0.76–1.48]
School education					
Low	Ref.				
Medium	0.95 [0.61–1.49]				
High	1.52 [1.00–2.31]				
Partnership					
No	Ref.				Ref.
Yes	**0.68 [0.51–0.91]**				0.80 [0.61–1.04]
Employment					
None	Ref.				Ref.
Part-time	**2.38 [1.54–3.67]**				**2.45 [1.66–3.60]**
Full-time	**2.40 [1.48–3.88]**				**2.08 [1.33–3.25]**
Lifestyle characteristics					
Smoking					
Current		**1.39 [1.01–1.93]**			1.16 [0.82–1.63]
Past		**1.57 [1.15–2.14]**			**1.46 [1.05–2.02]**
Never		Ref.			Ref.
E-cigarette use					
Current		**1.76 [1.15–2.69]**			**1.73 [1.11–2.70]**
Past		**1.64 [1.20–2.24]**			1.30 [0.92–1.83]
Never		Ref.			Ref.
Skin-related risk factors					
More than 40 naevi					
Yes			**1.43 [1.12–1.83]**		**1.32 [1.02–1.71]**
No			Ref.		Ref.
Family history of malignant melanoma					
Yes			**1.61 [1.15–2.26]**		1.16 [0.81–1.66]
No/don’t know			Ref.		Ref.
History of malignant melanoma					
Yes			1.55 [0.94–2.54]		
No			Ref.		
UV-related risk awareness					
“Each sunburn leaves a permanent damage in the skin”					
Rather agree				Ref.	
Rather disagree				0.906 [0.66–1.23]	
Don’t know				0.95 [0.45–2.00]	
“Regular use of tanning beds causes premature skin aging”					
Rather agree				Ref.	Ref.
Rather disagree				**1.42 [1.02–1.97]**	**1.41 [1.02–1.96]**
Don’t know				1.64 [0.82–3.28]	**1.97 [1.01–3.87]**
“Regular tanning bed use increases the skin cancer risk”					
Rather agree				Ref.	Ref.
Rather disagree				**3.71 [2.72–5.05]**	**3.20 [2.34–4.36]**
Don’t know				0.98 [0.47–2.04]	0.96 [0.46–2.01]
Nagelkerke’s r^2^	0.050	0.023	0.017	0.081	0.123
*n*	3317	3993	3993	3988	3749

*n* = 4000 individuals 16–65 years of age who participated in National Cancer Aid Monitoring in 2019; data is weighted by sex, age, education level and state of residence. Dependent variable: Tanning bed use during last 12 months (1 = yes, 0 = no); Regression models only included variables that were significant in bivariate analyses. Model I included sociodemographic characteristics, Model II included lifestyle characteristics, Model III included skin-related risk factors, Model IV included variables on UV-related risk awareness as independent variables. Model V included only variables that were significant in preceding regression model. Bold font: results with significance of *p* < 0.05; OR: Odds Ratio; 95% CI: 95% confidence interval, Ref.: Reference Category.

## Data Availability

The data presented in this study are available upon reasonable request from the corresponding author.
